# Influence of molecular marker type on estimating effective population size and other genetic parameters in a critically endangered parrot

**DOI:** 10.1002/ece3.11102

**Published:** 2024-03-24

**Authors:** George Olah, Robin S. Waples, Dejan Stojanovic

**Affiliations:** ^1^ Fenner School of Environment and Society Australian National University Canberra Australian Capital Territory Australia; ^2^ King's Forensics, Department of Analytical, Environmental and Forensic Sciences, Faculty of Life Sciences and Medicine King's College London London UK; ^3^ School of Aquatic and Fishery Sciences University of Washington Seattle Washington USA

**Keywords:** comparative study, effective population size, *Lathamus discolor*, molecular markers, population structure, swift parrot

## Abstract

Genetics is a fast‐moving field, and for conservation practitioners or ecologists, it can be bewildering. The choice of marker used in studies is fundamental; in the literature, preference has recently shifted from microsatellites to single nucleotide polymorphism (SNP) loci. Understanding how marker type affects estimates of population genetic parameters is important in the context of conservation, especially because the accuracy of estimates has a bearing on the actions taken to protect threatened species. We compare parameter estimates between seven microsatellites, 3761 SNP loci, and a random subset of 100 SNPs for the exact same 324 individual swift parrots, *Lathamus discolor*, and also use 457 additional samples from subsequent years to compare SNP estimates. Both marker types estimated a lower *H*_O_ than *H*_E_. We show that microsatellites and SNPs mainly indicate a lack of spatial genetic structure, except when a priori collection locations were used on the SNP data in a discriminant analysis of principal components (DAPC). The 100‐SNP subset gave comparable results to when the full dataset was used. Estimates of effective population size (*N*_e_) were comparable between markers when the same individuals were considered, but SNPs had narrower confidence intervals. This is reassuring because conservation assessments that rely on population genetic estimates based on a few microsatellites are unlikely to be nullified by the general shift toward SNPs in the literature. However, estimates between markers and datasets varied considerably when only adult samples were considered; hence, including samples of all age groups is recommended to be used when available. The estimated *N*_e_ was higher for the full SNP dataset (2010–2019) than the smaller comparison data (2010–2015), which might be a better reflection of the species status. The lower precision of microsatellites may not necessarily be a barrier for most conservation applications; however, SNPs will improve confidence limits, which may be useful for practitioners.

## Graphical abstract

Understanding how marker type affects estimates of population genetic parameters is important in the context of conservation, especially if the accuracy of estimates has a bearing on the actions taken to protect a species. We provide the first contrast of effective population size estimates between microsatellites and SNP markers for the exact same 324 individual swift parrots. Our results provide reassurance that, despite variation in the precision of different markers for estimating genetic parameters, conservation assessments using different approaches are comparably accurate.

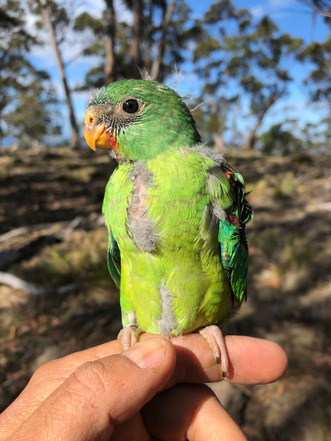

## 1 INTRODUCTION

Conservation practitioners are increasingly embracing the application of genetic tools and techniques (Frankham et al., 2010; Hendricks et al., 2018; Schweizer et al., 2021). However, molecular ecology is a fast‐moving field, and nonspecialists are faced with a bewildering array of marker types and analytical techniques that can be applied in different ways to answer the same questions. For example, microsatellites or short tandem repeats (STRs) have been the marker of choice for most population genetic studies for the last few decades (Olah, Smith, et al., 2021; Parker et al., 1998), but more recently, single nucleotide polymorphisms (SNPs) have become increasingly popular due to the increased genomic coverage, low genotyping error, lower costs, and faster turnaround associated with sequencing them in a massively parallel fashion (Anderson & Garza, 2006). Identifying a suitable marker is the first step for a conservation genetics project, and navigating the choice may be daunting for a nonspecialist (Allendorf et al., 2010; Benestan et al., 2016). This is especially so in the context of threatened species management, where estimates of population genetic parameters may determine which intervention approaches are applied.

A recent review of differences between marker types found that for most studies where the exact same individuals or populations were sequenced using both microsatellites and SNPs, population heterozygosity (*H*_O_ and/or *H*_E_) estimates are usually lower when SNPs are used, reflecting differences in methodology and approach between marker types (Sunde et al., 2020). Furthermore, fine‐scale patterns of genetic structure were more detectable and discerned in greater detail when using SNPs than microsatellites (Sunde et al., 2020). Highly polymorphic microsatellite markers are especially useful for threatened species where genetic variation has been greatly reduced (Hauser et al., 2021). On one hand, up‐front expenses to design and validate microsatellite markers for each species are often high, whereas prior genetic information is not required for many SNP methods. On the other hand, microsatellite markers are often applicable across closely related species with some ascertainment bias (Gebhardt & Waits, 2008; Olah et al., 2015), and rapidly accumulating genomic data makes the development of new markers faster and cost‐effective for many species (Abdelkrim et al., 2009). Still, studies that compare effective population size (*N*_e_) estimates between marker types remain relatively scarce (Athrey et al., 2018; Lemopoulos et al., 2019). Understanding the extent to which these patterns are representative across taxa is important both for studies of molecular ecology in general, as well as for species conservation, where genetic information is used for planning.

In this study, we use the Critically Endangered swift parrot *Lathamus discolor* as a model to contrast estimates of *N*_e_, observed and expected heterozygosity (*H*_O_ and *H*_E_), the inbreeding coefficient (*F*_IS_), and spatial structure using different, putatively neutral marker types on the exact same individuals. We previously used seven polymorphic microsatellite markers to show that this nomadic species is panmictic and lacks spatial structure across its Tasmanian breeding range (Stojanovic et al., 2018) and to estimate *N*_e_ (Olah, Stojanovic, et al., 2021). Here, we obtain SNPs for the same set of individual parrots to quantify the impact of marker type on the accuracy and precision of estimates. We then recalculate *H*_O_, *H*_E_, *F*_IS_, and *N*_e_, census population size (*N*), and spatial structure using SNP loci with an additional 4 years of data to evaluate the impact of larger sample sizes on these estimates. Finally, we assess the effect of the number of SNP markers on these estimates.

## 2 MATERIALS AND METHODS

All DNA samples were collected with approval from the Australian National University Animal Ethics and Experimentation Committee, and under scientific licenses from the Tasmanian Government. Our earlier studies comprised a sample of total 341 swift parrots: 310 nestlings from 93 nests and 31 adults collected between 2010 and 2015 (Olah, Stojanovic, et al., 2021; Stojanovic et al., 2018). These were added to an additional 4 years of data (2016–2019; *n* = 457 parrots) and provided to Diversity Arrays Technology Pty. Ltd. (DArT; Canberra, Australia) for DNA extraction, molecular sexing, and SNP genotyping using DartSeq™ protocols, combining complexity reduction based on restriction fragments from enzyme pairs, with next generation sequencing (NGS) technology (Jaccoud et al., 2001; Kilian et al., 2012). They successfully genotyped 781 individuals, including nestlings from 231 nests. We filtered SNPs using the *dartR* package in R (Gruber et al., 2018; R Core Team, 2022), based on a 0.99 reproducibility threshold, retaining one variant per sequence tag, variants without missing data (call rate threshold of 1), and a minimum minor allele frequency of 3%.

All analyses were repeated on two data subsets: (1) 324 individual parrots included in our previous analysis (Olah, Smith, et al., 2021) for which both microsatellites and SNPs were available (hereafter called the “comparison” dataset), and (2) the “full” dataset of 781 individuals. The comparison dataset highlights the effect of marker type on the magnitude and precision of each parameter, whereas the full dataset updates each parameter using SNPs on a larger sample of parrots. The comparison dataset is slightly smaller than that of our previous studies because we excluded 17 individual swift parrots from the earlier work whose SNP genotyping failed.

We calculated *H*_O_, *H*_E_, and *F*_IS_ in GenAlEx v.6.5 (Peakall & Smouse, 2012). It has been suggested that at least 2–11 times the number of SNP loci may be required for comparable resolution in genetic differentiation to microsatellites (Haasl & Payseur, 2011; Kalinowski, 2002; Morin et al., 2004). To test the importance of the number of SNPs on the accuracy and precision of estimates, we calculated the focal parameters for the full filtered SNP data set as well as a random subset of 100 SNPs. We visualized the relative position of individual samples grouped by a priori locations (Table S1) in the genetic space by conducting a principal coordinate analysis (PCoA) in GenAlEx v.6.5 (Peakall & Smouse, 2012). We examined genetic structure/admixture among individual genotypes first by Principal Component Analyses (PCA) and then by sparse non‐negative matrix factorization (SNMF) algorithms, both implemented in the *LEA* package in R (Frichot & François, 2015). The “snmf” function computes ancestries of each individual in chains of given clusters (*K* = 1–7 with 20 repetitions each). It estimates an entropy criterion that evaluates the quality of fit of the statistical model to the original data using a cross‐validation technique, where we blinded 10% of the data. Low cross‐entropy values reflect the number of most likely ancestral populations, which best explains the genotypic data. We kept the alpha parameter at the default. We also explored how the number of SNP loci influenced the patterns of genetic structure and repeated the admixture analysis on randomly selected subsets of 100, 500, 1000, and 2000 SNPs in the full dataset. Finally, we investigated any potential structure in the datasets by implementing discriminant analysis of principal components (DAPC) using the *adegenet* package in R (Jombart & Ahmed, 2011). This approach is sensitive to fine genetic differences among populations and identifies clusters where the within‐group component of variation is minimized compared to between‐group variation. First, we used the “find.Clusters” function (*K* = 1–10) retaining all principal components (PCs) and then obtained the number of PCs for DAPC by cross‐validation. We selected the lowest number of components with the greatest power of discrimination (60 for microsatellites, 100 for SNPs in the comparison dataset, and 200 for SNPs in the full dataset; Figure S1).

We followed our earlier approach (Olah, Stojanovic, et al., 2021) and used the linkage disequilibrium (LD) method (Waples, 2006, 2024; Waples & Do, 2010) implemented in the software NeEstimator v2.1 (Do et al., 2014) to estimate *N*_e_. Briefly, we used a threshold frequency of 0.02, assumed polygamy, and calculated 95% CIs for *N*_e_ by a jackknife‐across‐samples method (Jones et al., 2016). We calculated adjusted values of *N*_e_ (see formula in tab. 3 of Waples et al., 2014) by using AL (adult life span = 8 years), α (age at maturity = 2 years), and CVf (coefficient of variation of mean number of offspring for adult life span = 0). We used previously published data on life history parameters (Olah, Stojanovic, et al., 2021) to calculate the rate of *N*_e_/census population size (*N*) using the software AgeNe (Waples et al., 2011). We also calculated the weighted harmonic mean of *N*_e_ estimates based on microsatellites and SNPs, using the effective *df* for weights (Waples & Do, 2010). We calculated *N*_e_ and *N* estimates on samples in the comparison dataset (*n* = 324, of which *n*_adults_ = 29, *n*_nestlings_ = 295) and the full dataset (*n* = 781, of which *n*_adults_ = 44, *n*_nestlings_ = 737).

## 3 RESULTS

Filtering left us with 3761 SNP loci for analysis. We report *H*_O_, *H*_E_, and *F*_IS_ for the comparison and full datasets in Table 1. There was a large difference in estimates of heterozygosity between the two marker types, with microsatellites producing much higher absolute values. In contrast, the difference between markers in *F*_IS_ values was smaller, given the confidence intervals around the estimates. SNPs produced comparably high confidence results regardless of the data or marker subset used, although using fewer SNP loci resulted in lower *F*_IS_ values (Table 1).

**TABLE 1:** Estimates of *H*_O_, *H*_E_, and *F*_IS_ with standard errors in parentheses for the comparison (*n* = 324) and full (*n* = 781) datasets of swift parrots *Lathamus discolor*.

Dataset	Marker type	Number of markers	*H*_O_	*H*_E_	*F*_IS_
Comparison (2010–2015)	Microsatellites	7	0.678 (±0.075)	0.681 (±0.073)	0.007 (±0.012)
	SNPs	100	0.254 (±0.014)	0.258 (±0.014)	0.018 (±0.009)
	SNPs	3761	0.257 (±0.002)	0.263 (±0.002)	0.025 (±0.002)
Full (2010–2019)	SNPs	100	0.252 (±0.014)	0.256 (±0.014)	0.018 (±0.007)
	SNPs	3761	0.257 (±0.002)	0.263 (±0.002)	0.027 (±0.001)

**FIGURE 1:**
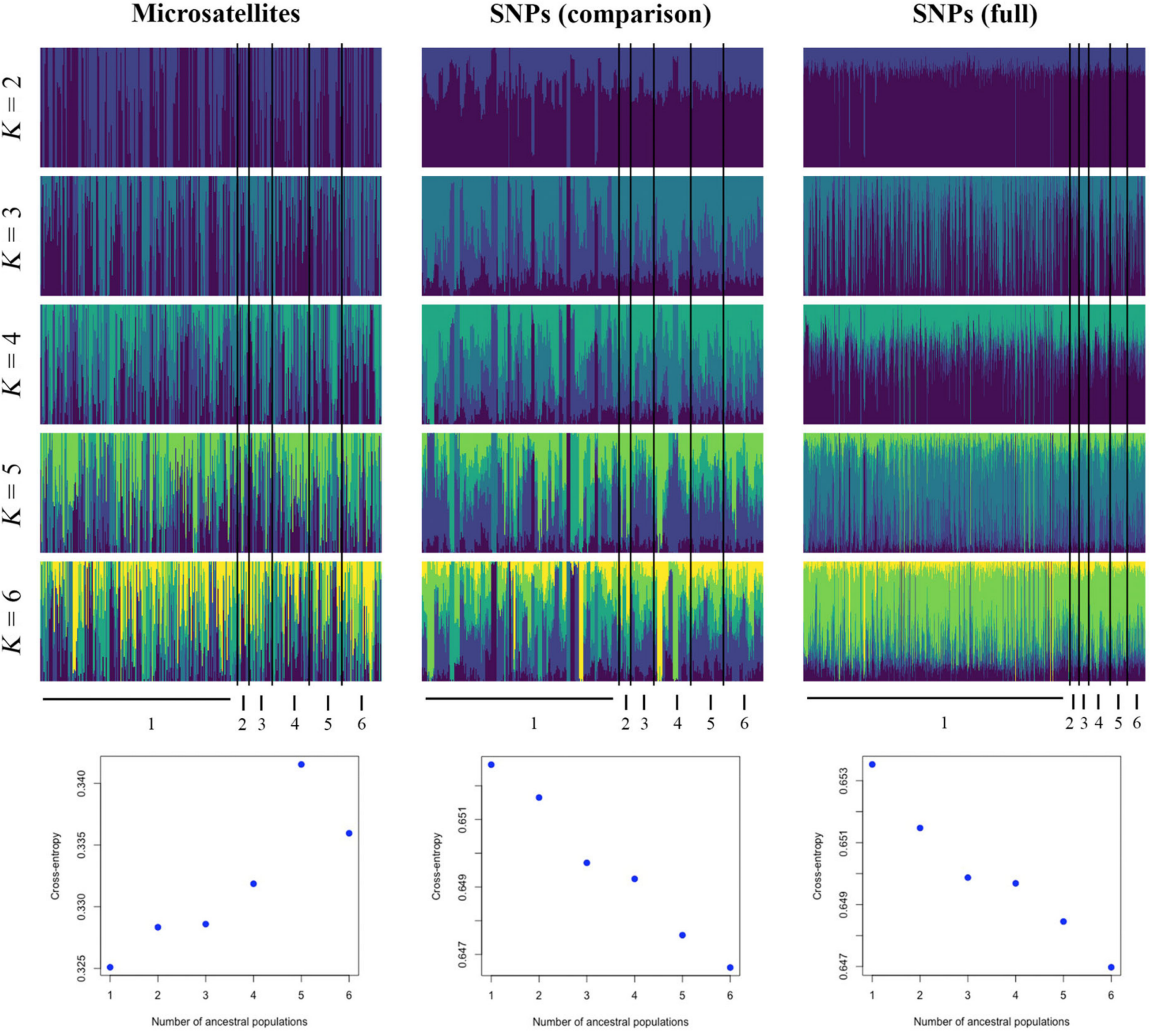
Admixture analyses with sparse non‐negative matrix factorization (SNMF) on the microsatellite and SNP datasets of swift parrots *Lathamus discolor*. Displayed are the individual ancestry coefficients for *K* = 2–6 clusters on top and the cross‐entropy plots below. Collection locations in Tasmania demark Bruny Island (1), Southern Forests (2), Meehan Range (3), Wielangta & Rheban (4), Buckland (5), and Eastern Tiers (6). The microsatellite and the comparison SNP datasets are based on the same 324 individuals (collected between 2010 and 2015), and the full SNP dataset is based on a total of 781 samples (spanning from 2010 to 2019).

**FIGURE 2:**
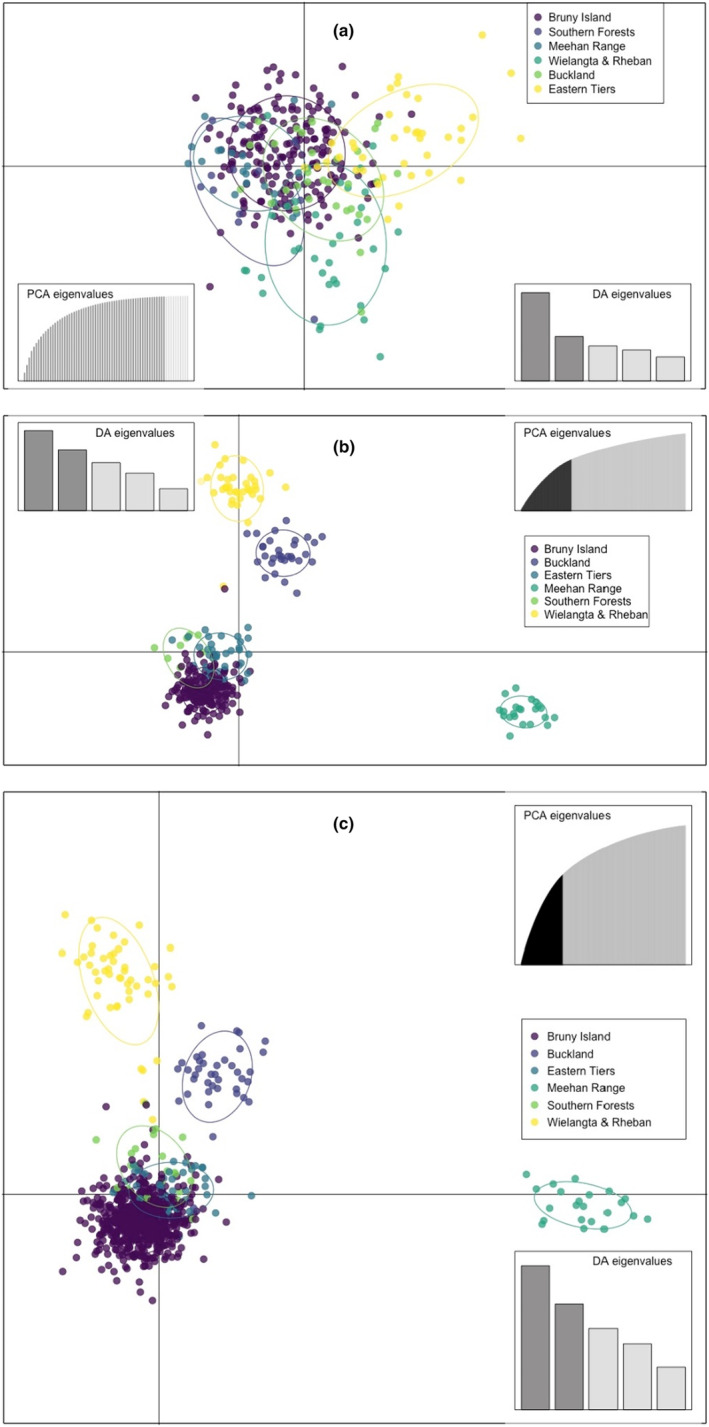
Grouping of swift parrot *Lathamus discolor* samples by discriminant analysis of principal components (DAPC) based on (a) microsatellite data, (b) SNP data from the same individuals (*n* = 324), and (c) SNP data from the full dataset (*n* = 781). Colors show collection locations (Table S1).

Population genetic structure could not be found regardless of marker type using PCoA (Figure S2), PCA (Figure S3), or the cluster analysis (Figure S3). Admixture analysis could not detect any genetic structure (Figure 1) regardless of the number of SNP loci used (Figure S4). However, when six a priori swift parrot collection locations were used (Table S1), DAPC successfully parsed out some of the locations using the SNP but not the microsatellite dataset (Figure 2 and Figure S5).

Estimates of *N*_e_ are reported in Table 2 for the comparison and full datasets. For the comparison dataset, relative to microsatellites, SNPs improved the precision of *N*_e_ estimates. The estimates derived from microsatellites and SNPs were comparable, but the microsatellites consistently resulted in lower estimates of *N*_e_. Neither marker could calculate finite upper confidence limits for adult parrots, where estimates between markers and datasets varied considerably. Based on the full dataset, estimates of *N*_e_ were double the weighted harmonic mean of the comparison dataset when all samples were considered (i.e. nestlings + adults, Table 2). Based on the adjusted *N*_e_ for the full dataset when all samples were pooled, we estimate the census population size of swift parrots was 498 individuals (CI: 469–530) over the decade when the samples were collected. When results from the total number of SNPs were compared to those from just 100 randomly selected SNP markers, *N*_e_ estimates were similar, except for the adult samples in the comparison dataset, where *N*_e_ estimates for the total SNPs were almost sixfold larger than that for the 100‐SNP‐subset (Table S2).

**TABLE 2:** Estimates of effective population size (*N*_e_) calculated with the linkage disequilibrium (LD) method for (1) a comparison dataset of *n* = 324 swift parrots genotyped using seven microsatellites (msat) and 3761 single nucleotide polymorphisms (SNPs), and (2) a full dataset of *n* = 781 individuals (between 2010 and 2019) based on the 3761 SNPs only.

Age	Comparison dataset						Full dataset (SNPs only)			
	*n*	*N*_e_ (msat)	*N*_e_ (SNPs)	*N*_e_ (HMw)	*N*_e_ (HMw adj)	*N* (adj)	*n*	*N*_e_	*N*_e_ (adj)	*N* (adj)
Adults	29	371 (23–∞)	1838 (455–∞)	1805 (419–∞)	1516 (352–∞)	2861 (664–∞)	44	193 (77–∞)	162 (65–∞)	306 (122–∞)
Nestlings	295	127 (66–301)	162 (149–177)	162 (147–177)	136 (124–149)	256 (234–281)	737	319 (302–339)	268 (253–284)	506 (478–537)
All	324	125 (67–270)	159 (146–173)	159 (144–174)	133 (121–146)	251 (229–276)	781	314 (296–334)	264 (249–281)	498 (469–530)

*Note*: We calculated the weighted harmonic mean (HMw) of the two estimates of *N*_e_ in the comparison dataset, the adjusted (adj) *N*_e_, and census population size (*N*) for adults, nestlings, and all samples. Parentheses show 95% CIs calculated by a jackknife‐across‐samples method.

## 4 DISCUSSION

For calculating common population genetic diversity metrics, SNPs had narrower confidence limits compared to microsatellites when applied to the exact same individual swift parrots (Table 1). For effective population size, the estimates made with SNPs fell within the confidence limits of the estimates based on only seven microsatellites, when nestlings or all samples were considered (Table 2). Using only microsatellite markers, we could not detect any genetic structure here (Figure 1) or in our previous study (Stojanovic et al., 2018), which is unsurprising due to the nomadism of the swift parrot (Webb et al., 2014). SNPs, however, were shown to recover finer population structure in the same individuals when compared to equivalent microsatellite data in fish (Jeffries et al., 2016), amphibians (Camacho‐Sanchez et al., 2020), and bats (Dufresnes et al., 2023). Here, only DAPC on the SNP dataset including a priori collection locations and many PCs meaningfully grouped the samples (Figure 2), although broader structure was not detected in the admixture analyses. Thus, both markers were useful, but consideration must be given to the level of resolution needed for a particular application and the underlying population genetic structure (Rosenberg et al., 2003; Thalamuthu et al., 2005).

The number of markers should also be taken into consideration. Other comparative studies have used more microsatellites (*n* = 15 (Weinman et al., 2015), 111 (Layton et al., 2020), 328 (Liu et al., 2005)) or fewer SNPs (*n* = 960 (Tokarska et al., 2009), 300 (Glover et al., 2010), 38 (Dann et al., 2013)), so we also tested random subsets of our SNP profile to be more comparable with our microsatellites. The outcomes of these smaller subsets were similar to the full SNP dataset (Table 1 and Figure 1). It has been shown with admixture analysis simulations that even if gene flow exists between populations, adding more markers should considerably raise power to detect structure (Haasl & Payseur, 2011). A study on two groups of a threatened fish could only find signs of introgression when using SNPs but not microsatellite data for the same individuals (Bradbury et al., 2015). In our admixture analyses, we could not detect population structure in the swift parrot with 100 or over 3000 SNPs (Figure S4). However, sampling from admixed populations might be misleading, as the signal of population structure can be diluted after several generations since admixture (Haasl & Payseur, 2011). Nevertheless, the DAPC analyses were able to identify some weak structure with the full SNP dataset (Figure 2 and Figure S5).

Both marker types estimated a lower *H*_O_ than *H*_E_. As expected, SNPs and microsatellites were less comparable for the absolute values of *H*_O_, *H*_E_, and *F*_IS_ (Table 1). Microsatellite markers are deliberately designed to target highly polymorphic regions of the genome, whereas the SNPs used in this study are not. Consequently, SNPs produced lower *H*_O_ and *H*_E_ estimates, and higher estimates of *F*_IS_ than microsatellites. This is consistent with other studies (Hauser et al., 2021; Liu et al., 2017), reflecting the design of the markers and the biased sampling of the genome. *H*_O_ and *H*_E_ are expected to be higher for multi‐allelic loci like microsatellites, even if we must assume that this heterozygosity is underestimated because of homoplasy (although homoplasy is also likely to affect biallelic markers). Thus, *F*_ST_ and *F*_IS_ estimates are generally smaller for microsatellites (Crates et al., 2019; Liu et al., 2017; Tereba & Konecka, 2020). The lower heterozygosity in SNPs however, can influence their power in paternity exclusion analyses (Morin et al., 2004), which are important tools in threatened species conservation (Heinsohn et al., 2019). SNP‐derived estimates of *H*_O_, *H*_E_, and *F*_IS_ were robust to sample size, producing nearly identical estimates for the comparison and full datasets (Table 1).

*N*_e_ estimates were higher for the full dataset (from 2010 to 2019) than the smaller comparison data (Table 2)—whether this is a biological effect or just a sampling artifact is unclear. When only adult swift parrots were considered (29 in the comparison and 44 in the full dataset), estimates between markers and datasets varied considerably (Table 2). When all SNPs were used with the scarce adult samples in the comparison dataset, it resulted in higher values of estimates compared to the 100‐SNP‐subset (Table S2). Such upward bias could emerge, for instance, when using just one sibling per nest and biasing the sampling toward more unrelated individuals (Waples & Anderson, 2017), but in our case we did not take this approach. In the full dataset, however, using solely adult samples depressed the *N*_e_ estimates, possibly due to nonrandom sampling because some families must have been overrepresented in the adult samples. The bioinformatic processing of SNPs could also be responsible for these discrepancies (O'Leary et al., 2018). Regardless, in accordance with previously published simulations and empirical data (Olah, Stojanovic, et al., 2021; Waples & Anderson, 2017), we caution against only using a small number of adult samples of unknown pedigrees or purging sibling samples from the dataset when estimating *N*_e_.

Swift parrots are critically endangered, and their conservation status hinges on estimations of population size, number of subpopulations, and trends in both over time and space (Webb et al., 2020). Thus, it is crucial to know if different markers yield results divergent enough to alter the interpretation of their conservation needs. Our earlier results of low *N*_e_, using a handful of microsatellite markers (Olah, Stojanovic, et al., 2021) were subject to debate within the swift parrot conservation community, and estimates of population size derived from expert opinion prevail in published conservation assessments (Webb et al., 2020). Although there are clear methodological artifacts on the precision of estimates shown here, microsatellites and SNPs are reassuringly comparable when larger number (nestlings) of samples are used (Table 2), supporting our earlier conclusions that the species has a smaller population than previously assumed.

Previous studies comparing microsatellite and SNP markers were conducted on fish (Bradbury et al., 2015; Jeffries et al., 2016; Lemopoulos et al., 2019; Sunde et al., 2020), amphibians (Camacho‐Sanchez et al., 2020), birds (Hauser et al., 2021; Zimmerman et al., 2020), and mammals (Dufresnes et al., 2023; López‐Bao et al., 2020; Szatmári et al., 2021). These found similar results regarding heterozygosity estimates to our current study, but mainly focused on detecting genetic structure among populations (e.g. Jeffries et al., 2016) or relatedness among individuals (e.g. Lemopoulos et al., 2019). Our work contributes to a growing number of studies that contrast the application of different markers to the exact same set of individuals, and our results are comparable to other taxa (Bradbury et al., 2015; Dufresnes et al., 2023; Hauser et al., 2021; López‐Bao et al., 2020; Szatmári et al., 2021). We advance knowledge further by comparing effective population size estimates between markers in threatened wildlife. The rapid advances in genetic tools, including genomics (Brandies et al., 2019), are likely to yield further detailed insights into the genetic traits of populations (Olah, Smith, et al., 2021). Practitioners can be confident however, that a shift toward SNPs in the general literature does not necessarily make existing knowledge from microsatellites obsolete for conservation purposes.

## AUTHOR CONTRIBUTIONS

**George Olah:** Conceptualization (equal); data curation (equal); formal analysis (lead); methodology (equal); software (equal); validation (equal); visualization (lead); writing – original draft (equal); writing – review and editing (lead). **Robin S. Waples:** Conceptualization (supporting); formal analysis (supporting); methodology (equal); software (lead); supervision (supporting); validation (supporting); writing – original draft (supporting); writing – review and editing (supporting). **Dejan Stojanovic:** Conceptualization (equal); data curation (equal); formal analysis (supporting); funding acquisition (lead); investigation (lead); methodology (supporting); project administration (lead); resources (lead); software (supporting); supervision (equal); visualization (supporting); writing – original draft (equal); writing – review and editing (equal).

## FUNDING INFORMATION

The project received funding from the Australian Government Department of Agriculture, Water and the Environment. G.O. was funded by Australian Research Council DECRA Fellowship (DE230100085).

## CONFLICT OF INTEREST STATEMENT

The authors declare no conflict of interest.

### OPEN RESEARCH BADGES

This article has earned Open Data and Open Materials badges. Data and materials are available at https://doi.org/10.6084/m9.figshare.21769493.

## BENEFITS GENERATED

A research collaboration was developed with scientists from the countries providing genetic samples; all collaborators are included as co‐authors. All DNA samples were collected with approval from the Australian National University Animal Ethics and Experimentation Committee and under a scientific license from the Tasmanian Government. The results of the research have been shared with the provider communities in Tasmania and the broader scientific community, and the research addresses a priority concern: the conservation of the Critically Endangered swift parrot. More broadly, our group (difficultbirds.com) is committed to international scientific partnerships, as well as institutional capacity building.

## Supporting information


Data S1:

## Data Availability

All genetic data with sample metadata are available in FigShare (https://doi.org/10.6084/m9.figshare.21769493).
