# Supplementary material for: Influence of molecular marker type on estimating effective population size and other genetic parameters in a critically endangered parrot
Source: Ecol Evol. 2024 Mar 24;14(3):e11102. doi: 10.1002/ece3.11102 (PMC10961163; doi:10.1002/ece3.11102)

Supporting Information

Table S1. Number of swift parrot *Lathamus discolor* samples used in the analyses, divided by marker types and *a priori* sampling locations (broadly from south to north).

**Location**	**Comparison dataset**	**New samples (SNPs only)**	**All samples**
	**(2010–2015)**	**(2016–2019)**	**(2010–2019)**
1. Bruny Island	187	422	609
2. Southern Forests	11	10	21
3. Meehan Range	22	0	22
4. Wielangta & Rheban	35	14	49
5. Buckland	31	8	39
6. Eastern Tiers	38	3	41
**Total**	**324**	**457**	**781**

Table S2. Estimates of effective population sizes (N_e) for (1) a comparison dataset of n = 324 swift parrots (between 2010–2015) genotyped using a random subset of 100 SNPs and all 3,761 SNPs, and (2) a full dataset of n = 781 individuals (between 2010–2019) based on the same SNPs. All N_e estimates were calculated with the linkage disequilibrium (LD) method for subsets of adults, nestlings, and all samples. Parentheses show 95% CIs calculated by a jackknife-across-samples method.

**Age**	**Comparison dataset (2010–2015)**			**Full dataset (2010–2019)**		
	n	***N*e (100 SNPs)**	***N*e (3,761 SNPs)**	***n***	***N*e (100 SNPs)**	***N*e (3,761 SNPs)**
**Adults**	29	326 (100–∞)	1,838 (455–∞)	44	180 (81–∞)	193 (77–∞)
**Nestlings**	295	169 (146–198)	162 (149–177)	737	330 (295–370)	319 (302–339)
**All**	324	158 (137–184)	159 (146–173)	781	318 (286–355)	314 (296–334)

Figure S1. Cross-validation plots for (A) seven microsatellites (n = 324), (B) 3,761 SNPs in the comparison dataset (n = 324), and (C) the same number of SNPs in the full dataset (n = 781) of swift parrots *Lathamus discolor* in our DAPC analysis using varying numbers of PCs as implemented in the *adegenet* package in R (Jombart & Ahmed, 2011).

(A) Microsatellites

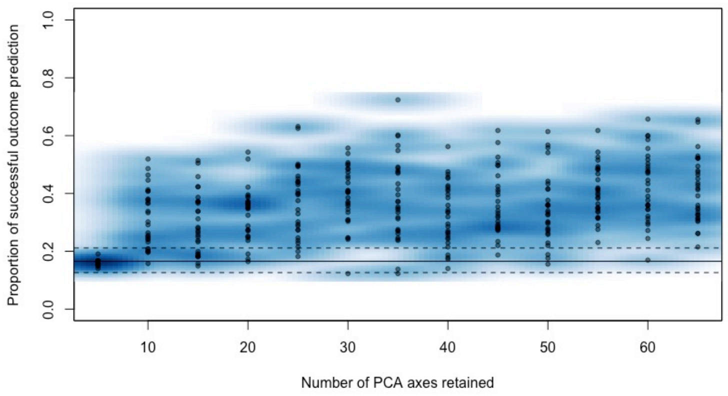

(B) SNPs (comparison dataset)

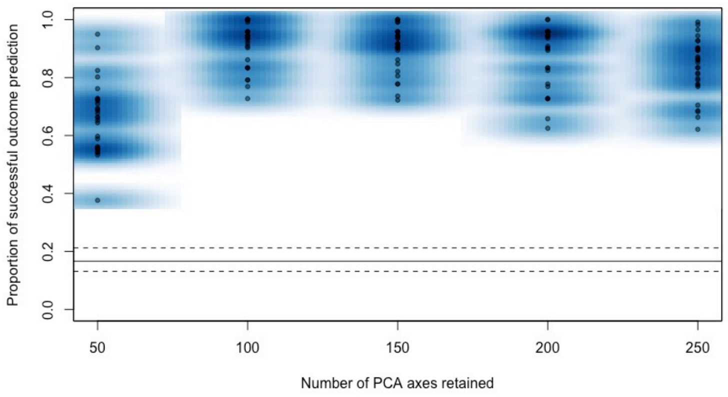

(C) SNPs (full dataset)

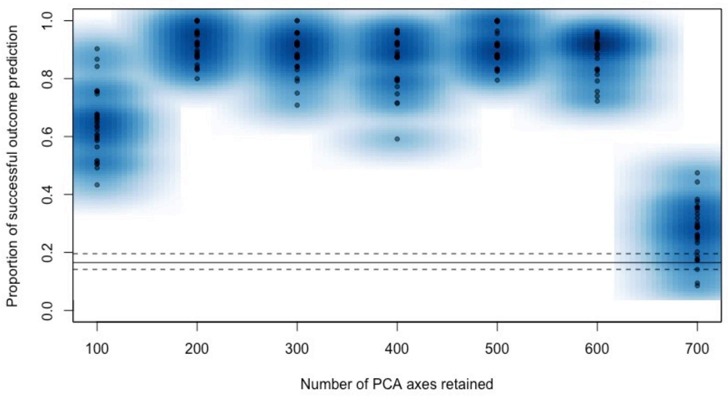

Figure S2. Principal coordinates analyses (PCoA) for our comparison sample of 324 swift parrots *Lathamus discolor* genotyped using (A) seven microsatellite and (B) 3,761 SNP loci. Percentage of variation explained by each coordinate is shown on the corresponding axes. Colours show six collection locations (Table S1).

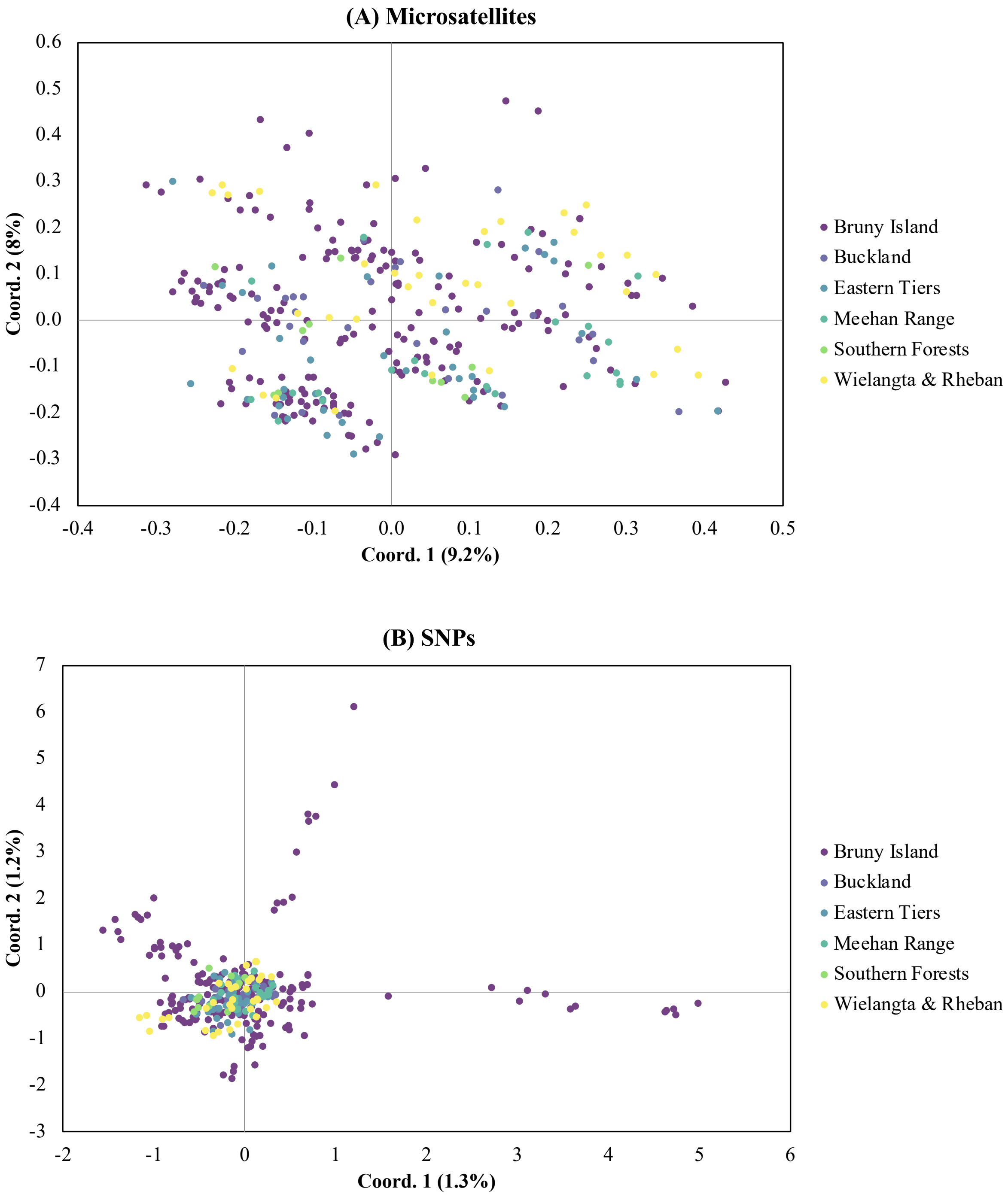

Figure S3. Principal component analyses (PCA; left) and cluster analyses (right) based on (A) seven microsatellites (*n* = 324), (B) 3,761 SNPs in the comparison dataset (*n* = 324), and (C) the same number of SNPs in the full dataset (*n* = 781) of swift parrots *Lathamus discolor*.

(A) Microsatellites

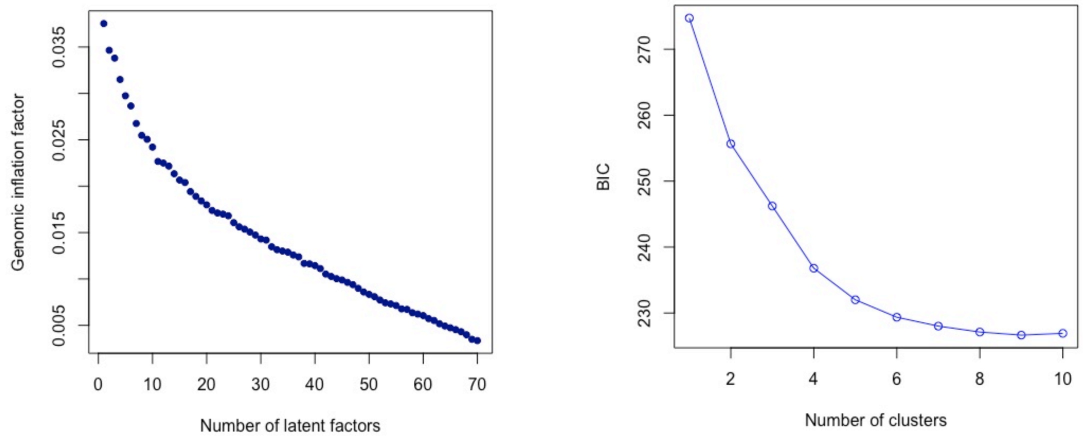

(B) SNPs (comparison dataset)

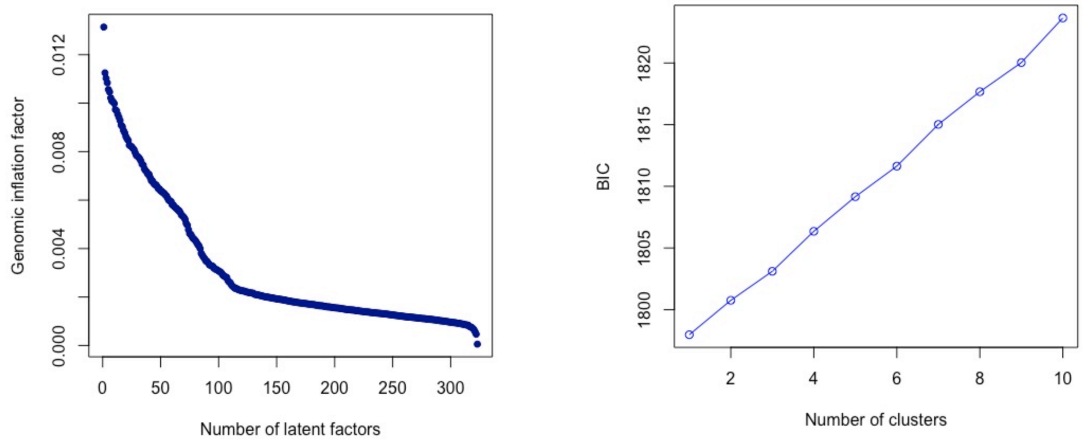

(C) SNPs (full dataset)

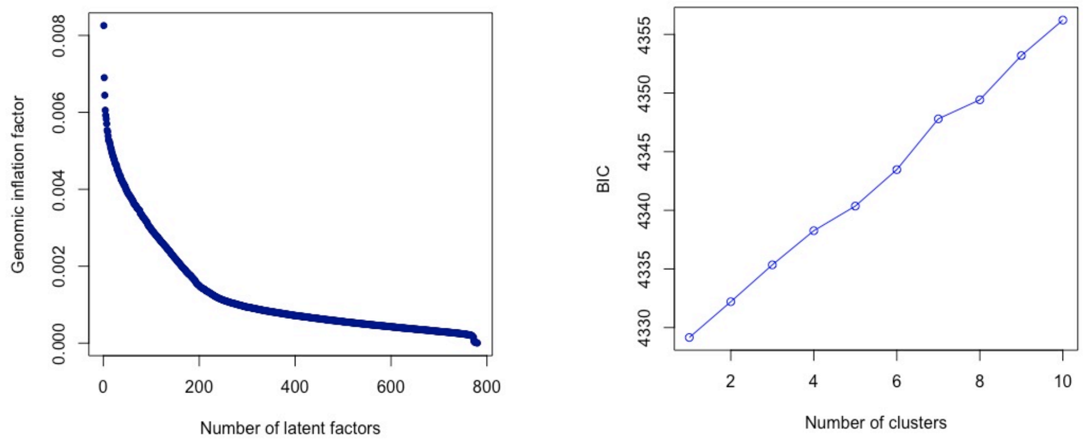

Figure S4. Admixture analyses with sparse non-negative matrix factorization (SNMF) on different subsets of SNPs of 781 swift parrots *Lathamus discolor*. Displayed are the individual ancestry coefficients for $K = 2–6$ clusters on top and the cross-entropy plots below. Collection locations in Tasmania demark Bruny Island (1), Southern Forests (2), Meehan Range (3), Wielangta & Rheban (4), Buckland (5), and Eastern Tiers (6).

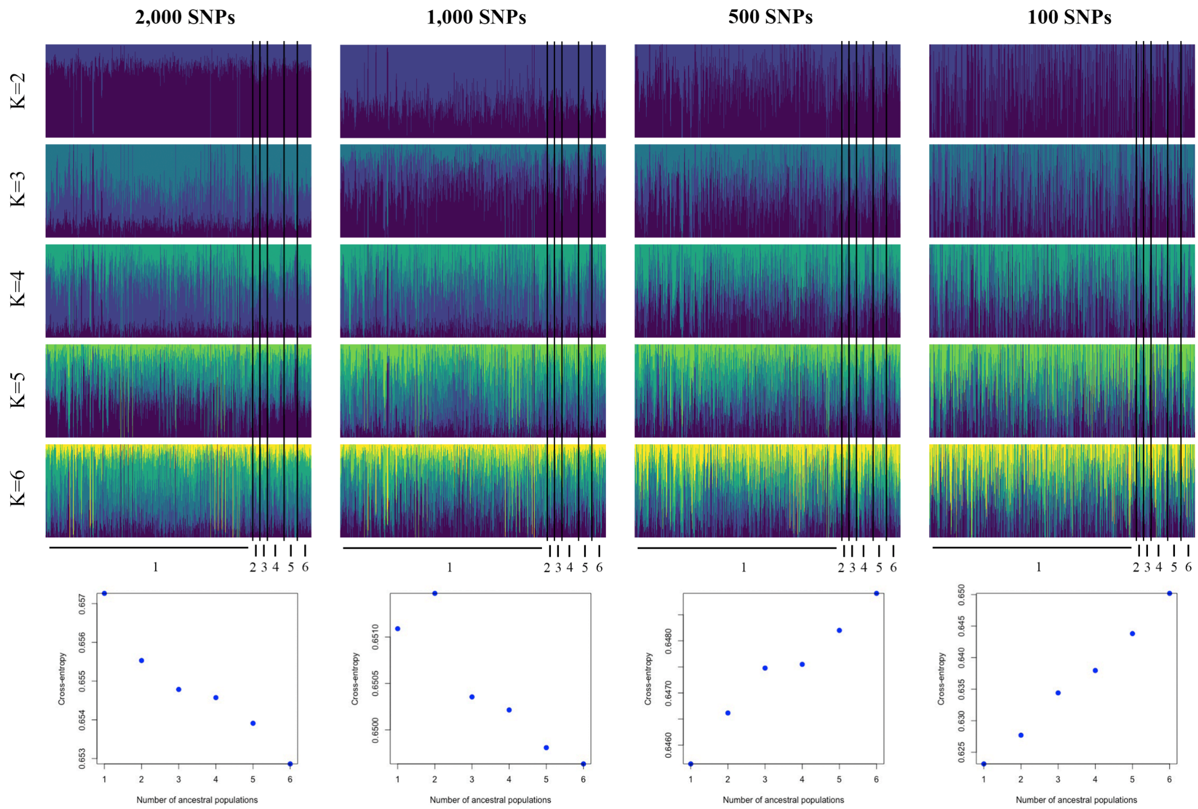

Figure S5. Grouping of swift parrot *Lathamus discolor* samples on discriminant analysis of principal components (DAPC) using the first discriminant function, based on (A) microsatellite data, (B) SNP data from the same individuals, and (C) SNP data from the full dataset. Colours show collection locations (Table S1).

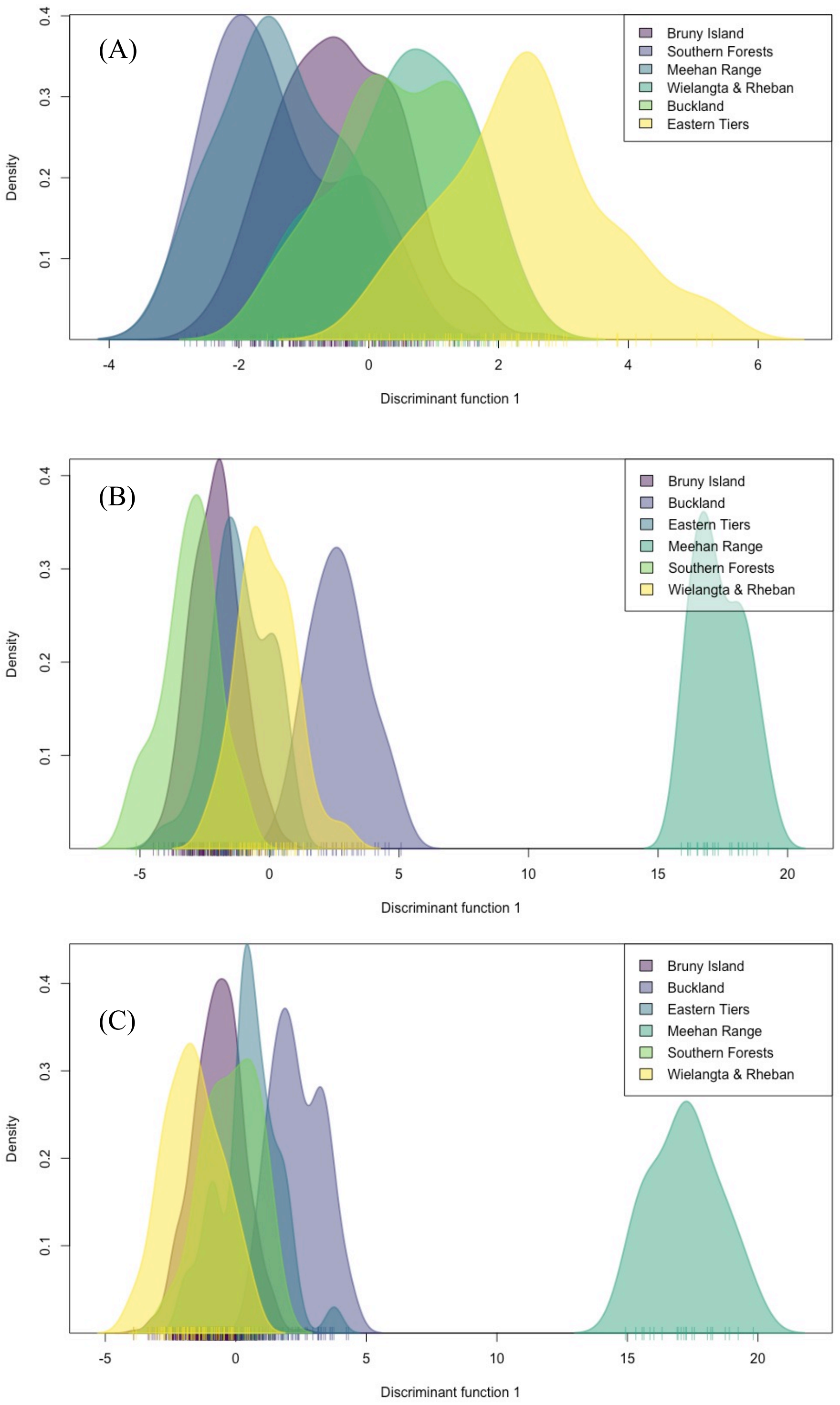

Supplement: Supplementary file 1 — Data S1: [file ECE3-14-e11102-s001.pdf]
